# Supplementary material for: Transgenerational Paternal Inheritance of TaCKX GFMs Expression Patterns Indicate a Way to Select Wheat Lines with Better Parameters for Yield-Related Traits
Source: Int J Mol Sci. 2023 May 3;24(9):8196. doi: 10.3390/ijms24098196 (PMC10179260; doi:10.3390/ijms24098196)
Supplement: Supplementary file 1 [file ijms-24-08196-s001.zip › ijms-2346183-supplementary.pdf]

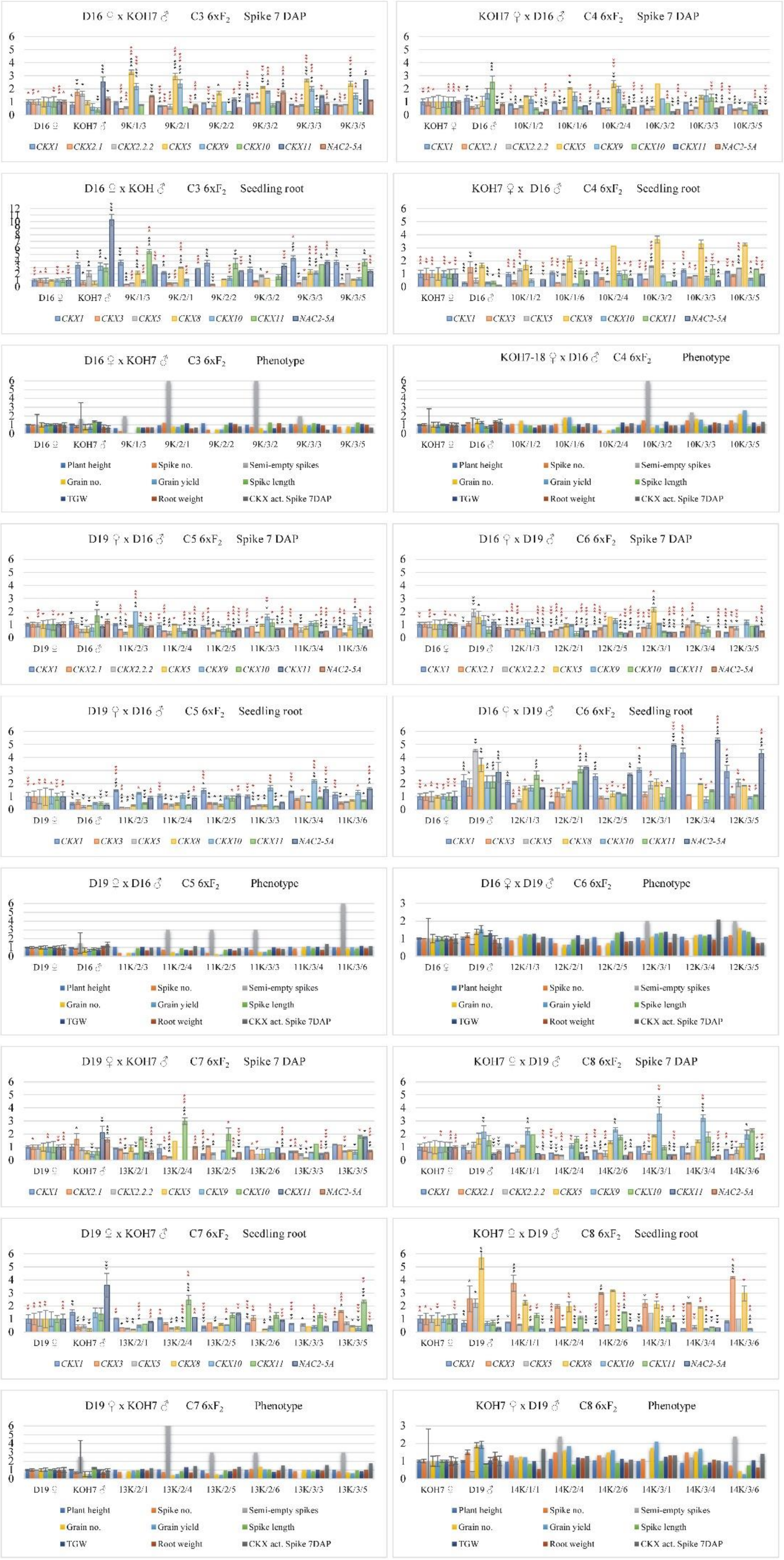
D16 ♀ x KOH7 ♂ C3 6xF2 Spike 7 DAP
D16 ♀ KOH7 ♂ 9K/1/3 9K/2/1 9K/2/2 9K/3/2 9K/3/3 9K/3/5
CKX1 CKX2.1 CKX2.2.2 CKX5 CKX9 CKX10 CKX11 NAC2-5A
KOH7 ♀ x D16 ♂ C4 6xF2 Spike 7 DAP
KOH7 ♀ D16 ♂ 10K/1/2 10K/1/6 10K/2/4 10K/3/2 10K/3/3 10K/3/5
CKX1 CKX2.1 CKX2.2.2 CKX5 CKX9 CKX10 CKX11 NAC2-5A
D16 ♀ x KOH ♂ C3 6xF2 Seedling root
D16 ♀ KOH7 ♂ 9K/1/3 9K/2/1 9K/2/2 9K/3/2 9K/3/3 9K/3/5
CKX1 CKX3 CKX5 CKX8 CKX10 CKX11 NAC2-5A
KOH7 ♀ x D16 ♂ C4 6xF2 Seedling root
KOH7 ♀ D16 ♂ 10K/1/2 10K/1/6 10K/2/4 10K/3/2 10K/3/3 10K/3/5
CKX1 CKX3 CKX5 CKX8 CKX10 CKX11 NAC2-5A
D16 ♀ x KOH7 ♂ C3 6xF2 Phenotype
D16 ♀ KOH7 ♂ 9K/1/3 9K/2/1 9K/2/2 9K/3/2 9K/3/3 9K/3/5
Plant height Spike no. Semi-empty spikes Grain no. Grain yield Spike length TGW Root weight CKX act. Spike 7DAP
KOH7-18 ♀ x D16 ♂ C4 6xF2 Phenotype
KOH7 ♀ D16 ♂ 10K/1/2 10K/1/6 10K/2/4 10K/3/2 10K/3/3 10K/3/5
Plant height Spike no. Semi-empty spikes Grain no. Grain yield Spike length TGW Root weight CKX act. Spike 7DAP
D19 ♀ x D16 ♂ C5 6xF2 Spike 7 DAP
D19 ♀ D16 ♂ 11K/2/3 11K/2/4 11K/2/5 11K/3/3 11K/3/4 11K/3/6
CKX1 CKX2.1 CKX2.2.2 CKX5 CKX9 CKX10 CKX11 NAC2-5A
D16 ♀ x D19 ♂ C6 6xF2 Spike 7 DAP
D16 ♀ D19 ♂ 12K/1/3 12K/2/1 12K/2/5 12K/3/1 12K/3/4 12K/3/5
CKX1 CKX2.1 CKX2.2.2 CKX5 CKX9 CKX10 CKX11 NAC2-5A
D19 ♀ x D16 ♂ C5 6xF2 Seedling root
D19 ♀ D16 ♂ 11K/2/3 11K/2/4 11K/2/5 11K/3/3 11K/3/4 11K/3/6
CKX1 CKX3 CKX5 CKX8 CKX10 CKX11 NAC2-5A
D16 ♀ x D19 ♂ C6 6xF2 Seedling root
D16 ♀ D19 ♂ 12K/1/3 12K/2/1 12K/2/5 12K/3/1 12K/3/4 12K/3/5
CKX1 CKX3 CKX5 CKX8 CKX10 CKX11 NAC2-5A
D19 ♀ x D16 ♂ C5 6xF2 Phenotype
D19 ♀ D16 ♂ 11K/2/3 11K/2/4 11K/2/5 11K/3/3 11K/3/4 11K/3/6
Plant height Spike no. Semi-empty spikes Grain no. Grain yield Spike length TGW Root weight CKX act. Spike 7DAP
D16 ♀ x D19 ♂ C6 6xF2 Phenotype
D16 ♀ D19 ♂ 12K/1/3 12K/2/1 12K/2/5 12K/3/1 12K/3/4 12K/3/5
Plant height Spike no. Semi-empty spikes Grain no. Grain yield Spike length TGW Root weight CKX act. Spike 7DAP
D19 ♀ x KOH7 ♂ C7 6xF2 Spike 7 DAP
D19 ♀ KOH7 ♂ 13K/2/1 13K/2/4 13K/2/5 13K/2/6 13K/3/3 13K/3/5
CKX1 CKX2.1 CKX2.2.2 CKX5 CKX9 CKX10 CKX11 NAC2-5A
KOH7 ♀ x D19 ♂ C8 6xF2 Spike 7 DAP
KOH7 ♀ D19 ♂ 14K/1/1 14K/2/4 14K/2/6 14K/3/1 14K/3/4 14K/3/6
CKX1 CKX2.1 CKX2.2.2 CKX5 CKX9 CKX10 CKX11 NAC2-5A
D19 ♀ x KOH7 ♂ C7 6xF2 Seedling root
D19 ♀ KOH7 ♂ 13K/2/1 13K/2/4 13K/2/5 13K/2/6 13K/3/3 13K/3/5
CKX1 CKX3 CKX5 CKX8 CKX10 CKX11 NAC2-5A
KOH7 ♀ x D19 ♂ C8 6xF2 Seedling root
KOH7 ♀ D19 ♂ 14K/1/1 14K/2/4 14K/2/6 14K/3/1 14K/3/4 14K/3/6
CKX1 CKX3 CKX5 CKX8 CKX10 CKX11 NAC2-5A
D19 ♀ x KOH7 ♂ C7 6xF2 Phenotype
D19 ♀ KOH7 ♂ 13K/2/1 13K/2/4 13K/2/5 13K/2/6 13K/3/3 13K/3/5
Plant height Spike no. Semi-empty spikes Grain no. Grain yield Spike length TGW Root weight CKX act. Spike 7DAP
KOH7 ♀ x D19 ♂ C8 6xF2 Phenotype
KOH7 ♀ D19 ♂ 14K/1/1 14K/2/4 14K/2/6 14K/3/1 14K/3/4 14K/3/6
Plant height Spike no. Semi-empty spikes Grain no. Grain yield Spike length TGW Root weight CKX act. Spike 7DAP

Figure S1. *TaCKX* GFM and *NAC2* expression profiles in 7 DAP spikes, seedling roots, and phenotypic traits in maternal parent, paternal parent, and their six F_2 progeny, from reciprocal cresses of D16 x KOH7 (C3) and KOH7 x D16 (C4); D19 x D16 (C5) and D16 x D19 (C6); D19 x KOH7 (C7) and KOH7 x D19 (C8). The data represent mean values with standard deviation. Black and red asterisks indicate statistical significance compared to the maternal parent or paternal parent respectively (* 0.05>p≥0.01, **0.01>p≥0.001, *** p<0.001) using the ANOVA test followed by the LSD post-hoc test (STATISTICA 10, StatSoft).

Table S1. Correlation coefficients between *TaCKX* GFMs and *NAC2* expression, CKX activity, and yield-related traits analysed separately for maternal plants and F_2, paternal plants and F_2 for each cross in four reciprocal crosses (C1 and C2; C3 and C4; C5 and C6; C7 and C8).

C1: S12B X S6C (M & F2; P & F2) C2: S6C x S12B (M & F2; P & F2)		*CKX1* Spike	*CKX2.1* Spike	*CKX2.2.2* Spike	*CKX5* Spike	*CKX9* (*10*) Spike	*CKX10* (*9*) Spike	*CKX11* (*3*) Spike	*NAC2-5A* Spike	*CKX1* Root	*CKX3* (*6*) Root	*CKX5* Root	*CKX8* (*11*) Root	*CKX10* (*9*) Root	*CKX11* (*3*) Root	*NAC2-5A* Root	CKX activity Root	CKX activity Spike	Plant height	Spike number	Empty spike number	Semi-empty spike number	Grain number	Grain yield	Spike length	TGW	Root wieght
CKX1 Spike	C1 (M + F_2)				0.73			0.63							-0.67			0.73					-0.61				
S12B x S6C	C1 (P + F_2)				0.39			-0.12							-0.43			0.58					-0.25				
CKX1 Spike	C2 (M + F_2)							0.16							-0.20			0.67			0.71						
S6C xS12B	C2 (P + F_2)							0.68							-0.60			0.87			0.55						
CKX2.1 Spike	C1 (M + F_2)			0.05														0.62		0.50						0.65	
S12B x S6C	C1 (P + F_2)			0.60														0.33		0.67						0.33	
CKX2.1 Spike	C2 (M + F_2)																										
S6C xS12B	C2 (P + F_2)																										
CKX2.2.2 Spike	C1 (M + F_2)																	0.37		0.65						-0.05	
S12B x S6C	C1 (P + F_2)																	0.61		0.74						0.69	
CKX2.2.2 Spike	C2 (M + F_2)											0.60	0.64	0.87						0.73							
S6C xS12B	C2 (P + F_2)											0.63	0.56	0.74						0.14							
CKX5 Spike	C1 (M + F_2)					0.50		0.68		-0.61	-0.33	0.29	0.22		-0.64	-0.37						0.62	-0.69				0.68
S12B x S6C	C1 (P + F_2)					0.76		0.80		-0.78	-0.61	0.66	0.63		0.01	-0.61						0.90	-0.32				0.38
CKX5 Spike	C2 (M + F_2)												-0.02														
S6C xS12B	C2 (P + F_2)												-0.60														
CKX9 (*10*) Spike	C1 (M + F_2)							0.38		-0.08												0.29					
S12B x S6C	C1 (P + F_2)							0.72		-0.66												0.66					
CKX9 (*10*) Spike	C2 (M + F_2)							0.60		-0.72												0.71			0.71		
S6C xS12B	C2 (P + F_2)							0.49		-0.21												0.50			0.42		
CKX10 (*9*) Spike	C1 (M + F_2)							0.75	0.83				0.61			-0.18										0.73	
S12B x S6C	C1 (P + F_2)							0.75	0.59				0.69			-0.69										0.13	
CKX10 (*9*) Spike	C2 (M + F_2)																										
S6C xS12B	C2 (P + F_2)																										
CKX11 (*3*) Spike	C1 (M + F_2)								0.79	-0.26			0.88		-0.71					-0.71		0.02		-0.65			0.65
S12B x S6C	C1 (P + F_2)								0.28	-0.69			0.86		0.07					-0.02		0.76		-0.15			0.32
CKX11 (*3*) Spike	C2 (M + F_2)									-0.83	-0.69		0.73	0.62		-0.29		0.04	0.83						0.64		
S6C xS12B	C2 (P + F_2)									-0.51	-0.33		0.62	0.35		0.67		0.60	0.25						0.49		
NAC2-5A Spike	C1 (M + F_2)										-0.32		0.66			-0.65								-0.66	-0.71	0.70	0.82
S12B x S6C	C1 (P + F_2)										-0.78		0.61			-0.88								-0.39	-0.55	0.50	0.33
NAC2-5A Spike	C2 (M + F_2)															-0.68											-0.60
S6C xS12B	C2 (P + F_2)															-0.63											0.35
CKX1 Root	C1 (M + F_2)										0.70			0.73		0.79	-0.70										-0.63
S12B x S6C	C1 (P + F_2)										0.46			-0.04		0.34	-0.40										-0.57
CKX1 Root	C2 (M + F_2)																		-0.81								
S6C xS12B	C2 (P + F_2)																		-0.25								
CKX3 (*6*) Root	C1 (M + F_2)											0.63		0.91		0.89											-0.63
S12B x S6C	C1 (P + F_2)											-0.17		0.25		0.93											-0.30
CKX3 (*6*) Root	C2 (M + F_2)												-0.70		-0.73	0.74							0.03	-0.01			
S6C xS12B	C2 (P + F_2)												0.17		-0.35	-0.15							0.61	0.66			
CKX5 Root	C1 (M + F_2)												0.74	0.58								-0.77					
S12B x S6C	C1 (P + F_2)												0.80	0.85								0.00					
CKX5 Root	C2 (M + F_2)												0.32						-0.21								
S6C xS12B	C2 (P + F_2)												0.60						-0.69								
CKX8 (*11*) Root	C1 (M + F_2)															-0.08	-0.48										
S12B x S6C	C1 (P + F_2)															-0.76	-0.76										
CKX8 (*11*) Root	C2 (M + F_2)													0.84	0.86	-0.64						-0.26					
S6C xS12B	C2 (P + F_2)													0.57	-0.28	0.19						-0.73					
CKX10 (*9*) Root	C1 (M + F_2)															0.88	-0.62					-0.69					-0.71
S12B x S6C	C1 (P + F_2)															0.01	-0.55					0.05					-0.54
CKX10 (*9*) Root	C2 (M + F_2)														0.75									0.63			
S6C xS12B	C2 (P + F_2)														0.26									0.53			
CKX11 (*3*) Root	C1 (M + F_2)																-0.22			0.75			0.88	0.84			-0.50
S12B x S6C	C1 (P + F_2)																-0.71			0.46			0.65	0.62			-0.71
CKX11 (*3*) Root	C2 (M + F_2)															-0.73		-0.63	0.44								
S6C xS12B	C2 (P + F_2)															-0.70		-0.62	0.61								
NAC2-5A Root	C1 (M + F_2)																										-0.74
S12B x S6C	C1 (P + F_2)																										-0.19
NAC2-5A Root	C2 (M + F_2)																	0.62		-0.45							
S6C xS12B	C2 (P + F_2)																	0.69		-0.64							
CKX activity Root	M + F2																	0.19									
S12B x S6C	P + F2																	0.64									
CKX activity Root	M + F2																						-0.54			0.91	
	P + F2																						-0.68			0.95	
CKX activity Spike	M + F2																			0.12							
S12B x S6C	P + F2																			0.70							
CKX activity Spike	M + F2																										
	P + F2																										
Plant height	M + F2																			0.55							
S12B x S6C	P + F2																			0.69							
Plant height	M + F2																								0.71		
S6C xS12B	P + F2																								0.35		
Spike number	M + F2																						0.52	0.70	-0.16		
S12B x S6C	P + F2																						0.76	0.82	0.69		
Spike number	M + F2																						0.85	0.84			
S6C xS12B	P + F2																						0.78	0.78			
Empty spike number	M + F2																										
S12B x S6C	P + F2																										
Empty spike number	M + F2																					-0.17					
S6C xS12B	P + F2																					0.60					
Semi-empty spike numbe	M + F2																								-0.60		
S12B x S6C	P + F2																								-0.27		
Semi-empty spike numbe	M + F2																										
S6C xS12B	P + F2																										
Grain number	M + F2																							0.95	0.60		-0.61
S12B x S6C	P + F2																							0.99	0.84		-0.60
Grain number	M + F2																							0.99		-0.57	
S6C xS12B	P + F2																							0.98		-0.74	
Grain yield	M + F2																								0.75		
S12B x S6C	P + F2																								0.81		
Grain yield	M + F2																										
S6C xS12B	P + F2																										
Spike length	M + F2																										
S12B x S6C	P + F2																										
TGW	M + F2																										
S12B x S6C	P + F2																										
Root weight	M + F2																										
S12B x S6C	P + F2																										

C3: D16 x KOH7 (M & F2; P & F2) C4: KOH7 x D16 (M & F2; P & F2)		*CKX1* Spike	*CKX2.1* Spike	*CKX2.2.2* Spike	*CKX5* Spike	*CKX9* (*10*) Spike	*CKX10* (*9*) Spike	*CKX11* (*3*) Spike	*NAC2-5A* Spike	*CKX1* Root	*CKX3* (*6*) Root	*CKX5* Root	*CKX8* (*11*) Root	*CKX10* (*9*) Root	*CKX11* (*3*) Root	*NAC2-5A* Root	CKX activity Root	CKX activity Spike	Plant height	Spike number	Empty spike number	Semi-empty spike number	Grain number	Grain yield	Spike length	TGW	Root wieght
CKX1 Spike	C3 (M + F_2)						0.44		0.78																		0.71
D16 x KOH7	C3 (P + F_2)						0.64		0.41																		0.57
CKX1 Spike	C4 (M + F_2)		0.76			0.74	0.87		-0.36	-0.70															0.66		
KOH7 x D16	C4 (P + F_2)		0.55			0.32	0.52		0.71	-0.46															0.24		
CKX2.1 Spike	C3 (M + F_2)			0.75	-0.73		0.33			-0.47	0.85		0.76		-0.69			0.60					0.65	0.68	0.54		
D16 x KOH7	C3 (P + F_2)			0.88	-0.59		0.66			-0.66	0.93		0.70		-0.68			0.38					0.59	0.47	0.63		
CKX2.1 Spike	C4 (M + F_2)			0.71		0.86	0.83	-0.22		-0.68	0.68					-0.60											0.06
KOH7 x D16	C4 (P + F_2)			0.63		0.66	0.59	-0.65		-0.57	0.59					-0.61											0.63
CKX2.2.2 Spike	C3 (M + F_2)				-0.75		0.52			-0.44	0.60				-0.66								0.51	0.60	0.45		
D16 x KOH7	C3 (P + F_2)				-0.53		0.83			-0.71	0.79				-0.69								0.60	0.50	0.64		
CKX2.2.2 Spike	C4 (M + F_2)						0.78			-0.70	0.59		0.68	-0.53		-0.87			-0.32							-0.82	0.37
KOH7 x D16	C4 (P + F_2)						0.59			-0.65	0.61		0.64	-0.61		-0.89			-0.71							-0.78	0.61
CKX5 Spike	C3 (M + F_2)					0.75			0.59		-0.70				0.62	0.77		-0.79									
D16 x KOH7	C3 (P + F_2)					0.44			0.67		-0.48				0.42	0.64		-0.67									
CKX5 Spike	C4 (M + F_2)							0.48			-0.18		-0.37		-0.69			-0.48									
KOH7 x D16	C4 (P + F_2)							0.61			-0.65		-0.60		-0.16			-0.63									
CKX9 (*10*) Spike	C3 (M + F_2)															0.60						0.60					
D16 x KOH7	C3 (P + F_2)															0.62						0.31					
CKX9 (*10*) Spike	C4 (M + F_2)						0.73					-0.66				-0.55											
KOH7 x D16	C4 (P + F_2)						0.19					-0.76				-0.62											
CKX10 (*9*) Spike	C3 (M + F_2)									-0.25						0.68											
D16 x KOH7	C3 (P + F_2)									-0.69						0.29											
CKX10 (*9*) Spike	C4 (M + F_2)									-0.65	0.64		0.85												0.58		0.19
KOH7 x D16	C4 (P + F_2)									0.20	0.47		0.67												-0.11		0.60
CKX11 (*3*) Spike	C3 (M + F_2)											0.79	-0.75									-0.51					0.56
D16 x KOH7	C3 (P + F_2)											0.74	-0.75									-0.64					0.70
CKX11 (*3*) Spike	C4 (M + F_2)																										
KOH7 x D16	C4 (P + F_2)																										
NAC2-5A Spike	C3 (M + F_2)												-0.64			0.63											0.44
D16 x KOH7	C3 (P + F_2)												-0.53			0.64											0.58
NAC2-5A Spike	C4 (M + F_2)																										
KOH7 x D16	C4 (P + F_2)																										
CKX1 Root	C3 (M + F_2)										-0.37		-0.50		0.67								-0.30		-0.16		
D16 x KOH7	C3 (P + F_2)										-0.76		-0.60		0.71								-0.63		-0.60		
CKX1 Root	C4 (M + F_2)										-0.83		-0.53			0.66											
KOH7 x D16	C4 (P + F_2)										-0.61		-0.60			0.65											
CKX3 (*6*) Root	C3 (M + F_2)												0.67		-0.80			0.38					0.74	0.65	0.69		
D16 x KOH7	C3 (P + F_2)												0.65		-0.76			0.62					0.85	0.73	0.89		
CKX3 (*6*) Root	C4 (M + F_2)											-0.51	0.83			-0.66		0.70									0.69
KOH7 x D16	C4 (P + F_2)											-0.61	0.90			-0.63		0.45									0.74
CKX5 Root	C3 (M + F_2)																										0.72
D16 x KOH7	C3 (P + F_2)																										0.75
CKX5 Root	C4 (M + F_2)															0.63		-0.67		0.69							
KOH7 x D16	C4 (P + F_2)															0.67		-0.49		0.68							
CKX8 (*11*) Root	C3 (M + F_2)																			0.56			0.71	0.67			-0.41
D16 x KOH7	C3 (P + F_2)																			0.66			0.65	0.60			-0.66
CKX8 (*11*) Root	C4 (M + F_2)															-0.88		0.70									0.60
KOH7 x D16	C4 (P + F_2)															-0.83		0.65									0.66
CKX10 (*9*) Root	C3 (M + F_2)															0.60		-0.57									
D16 x KOH7	C3 (P + F_2)															0.50		-0.66									
CKX10 (*9*) Root	C4 (M + F_2)																									0.72	
KOH7 x D16	C4 (P + F_2)																									0.67	
CKX11 (*3*) Root	C3 (M + F_2)																		-0.57				-0.65		-0.84		
D16 x KOH7	C3 (P + F_2)																		-0.64				-0.67		-0.84		
CKX11 (*3*) Root	C4 (M + F_2)																										
KOH7 x D16	C4 (P + F_2)																										
NAC2-5A Root	C3 (M + F_2)																	-0.68									
D16 x KOH7	C3 (P + F_2)																	-0.70									
NAC2-5A Root	C4 (M + F_2)																		0.26							0.68	-0.63
KOH7 x D16	C4 (P + F_2)																		0.63							0.60	-0.67
CKX activity Root	M + F2																										
	P + F2																										
CKX activity Spike	M + F2																										
D16 x KOH7	P + F2																										
CKX activity Spike	M + F2																									-0.60	0.61
KOH7 x D16	P + F2																									-0.53	0.33
Plant height	M + F2																						0.61	0.32	0.60	0.59	
D16 x KOH7	P + F2																						0.64	0.67	0.60	0.66	
Plant height	M + F2																						0.67	0.64	0.64		
KOH7 x D16	P + F2																						0.57	0.62	0.44		
Spike number	M + F2																						0.79	0.70			
D16 x KOH7	P + F2																						0.77	0.77			
Spike number	M + F2																					0.74	0.69	0.71			
KOH7 x D16	P + F2																					0.61	0.67	0.70			
Empty spike number	M + F2																										
D16 x KOH7	P + F2																										
Empty spike number	M + F2																										
KOH7 x D16	P + F2																										
Semi-empty spike numbe	M + F2																									-0.64	
D16 x KOH7	P + F2																									-0.47	
Semi-empty spike numbe	M + F2																										
KOH7 x D16	P + F2																										
Grain number	M + F2																							0.96	0.65	0.65	
D16 x KOH7	P + F2																							0.98	0.83	0.43	
Grain number	M + F2																							0.90	0.80		
KOH7 x D16	P + F2																							0.94	0.77		
Grain yield	M + F2																								0.54	0.79	
D16 x KOH7	P + F2																								0.76	0.59	
Grain yield	M + F2																								0.52		
KOH7 x D16	P + F2																								0.59		
Spike length	M + F2																										
D16 x KOH7	P + F2																										
TGW	M + F2																										
D16 x KOH7	P + F2																										
Root weight	M + F2																										
D16 x KOH7	P + F2																										

C5: D19 x D16 (M & F2; P & F2) C6: D16 x D19 (M & F2; P & F2)		*CKX1* Spike	*CKX2.1* Spike	*CKX2.2.2* Spike	*CKX5* Spike	*CKX9* (*10*) Spike	*CKX10* (*9*) Spike	*CKX11* (*3*) Spike	*NAC2-5A* Spike	*CKX1* Root	*CKX3* (*6*) Root	*CKX5* Root	*CKX8* (*11*) Root	*CKX10* (*9*) Root	*CKX11* (*3*) Root	*NAC2-5A* Root	CKX activity Root	CKX activity Spike	Plant height	Spike number	Empty spike number	Semi-empty spike number	Grain number	Grain yield	Spike length	TGW	Root wieght
CKX1 Spike	C5 (M + F_2)			-0.02					0.72	0.75		-0.73							-0.63				-0.70	-0.66			
D19 x D16	C5 (P + F_2)			0.68					0.91	0.08		-0.54							-0.27				-0.02	-0.05			
CKX1 Spike	C6 (M + F_2)							0.36	0.75		-0.58					-0.70			-0.67		0.67			-0.36			
D16 x D19	C6 (P + F_2)							0.68	0.25		-0.84					-0.72			-0.46		0.50			-0.73			
CKX2.1 Spike	C5 (M + F_2)			0.70				-0.67														-0.11					-0.03
D19 x D16	C5 (P + F_2)			0.91				-0.62														-0.64					0.68
CKX2.1 Spike	C6 (M + F_2)			0.87																0.39				0.58			0.71
D16 x D19	C6 (P + F_2)			0.58																0.58				0.40			0.25
CKX2.2.2 Spike	C5 (M + F_2)							-0.41							0.39												
D19 x D16	C5 (P + F_2)							-0.67							0.65												
CKX2.2.2 Spike	C6 (M + F_2)				-0.57																						0.78
D16 x D19	C6 (P + F_2)				-0.61																						0.70
CKX5 Spike	C5 (M + F_2)								0.68				-0.71	0.70	-0.91			-0.40							-0.61		
D19 x D16	C5 (P + F_2)								-0.04				-0.77	0.66	-0.87			-0.64							-0.31		
CKX5 Spike	C6 (M + F_2)							0.70	0.28			0.91							-0.71			0.62					
D16 x D19	C6 (P + F_2)							0.35	0.77			0.82							-0.77			0.62					
CKX9 (*10*) Spike	C5 (M + F_2)							0.12					-0.53														
D19 x D16	C5 (P + F_2)							0.66					-0.64														
CKX9 (*10*) Spike	C6 (M + F_2)										-0.61	0.74				-0.71		-0.70									
D16 x D19	C6 (P + F_2)										-0.71	0.78				-0.71		-0.81									
CKX10 (*9*) Spike	C5 (M + F_2)																				0.40						
D19 x D16	C5 (P + F_2)																				0.67						
CKX10 (*9*) Spike	C6 (M + F_2)							0.20													0.58						
D16 x D19	C6 (P + F_2)							0.61													0.31						
CKX11 (*3*) Spike	C5 (M + F_2)									-0.64			-0.31												0.66		
D19 x D16	C5 (P + F_2)									-0.58			-0.66												-0.18		
CKX11 (*3*) Spike	C6 (M + F_2)												0.20														
D16 x D19	C6 (P + F_2)												0.90														
NAC2-5A Spike	C5 (M + F_2)									0.68	-0.65				-0.77										-0.78		-0.60
D19 x D16	C5 (P + F_2)									0.01	-0.59				0.04										0.18		0.30
NAC2-5A Spike	C6 (M + F_2)																				0.65						
D16 x D19	C6 (P + F_2)																				0.48						
CKX1 Root	C5 (M + F_2)																		-0.61	-0.62							-0.65
D19 x D16	C5 (P + F_2)																		-0.50	-0.66							-0.17
CKX1 Root	C6 (M + F_2)													-0.77	-0.72												
D16 x D19	C6 (P + F_2)													-0.52	-0.54												
CKX3 (*6*) Root	C5 (M + F_2)															0.61							0.68	0.65			
D19 x D16	C5 (P + F_2)															0.46							0.71	0.66			
CKX3 (*6*) Root	C6 (M + F_2)															0.60											
D16 x D19	C6 (P + F_2)															0.74											
CKX5 Root	C5 (M + F_2)															0.66				0.62			0.73	0.67			
D19 x D16	C5 (P + F_2)													0.62		0.48				0.58			0.35	0.38			
CKX5 Root	C6 (M + F_2)												0.25	-0.62					-0.79			0.68					
D16 x D19	C6 (P + F_2)												0.85	-0.61					-0.63			0.44					
CKX8 (*11*) Root	C5 (M + F_2)													-0.64	0.66												
D19 x D16	C5 (P + F_2)													-0.75	0.60												
CKX8 (*11*) Root	C6 (M + F_2)																										
D16 x D19	C6 (P + F_2)																										
CKX10 (*9*) Root	C5 (M + F_2)														-0.72												
D19 x D16	C5 (P + F_2)														-0.44												
CKX10 (*9*) Root	C6 (M + F_2)														0.70												
D16 x D19	C6 (P + F_2)														0.01												
CKX11 (*3*) Root	C5 (M + F_2)																	0.70							0.72		0.63
D19 x D16	C5 (P + F_2)																	0.83							0.15		0.01
CKX11 (*3*) Root	C6 (M + F_2)																										
D16 x D19	C6 (P + F_2)																										
NAC2-5A Root	C5 (M + F_2)																		0.60	0.61			0.75	0.79		0.57	
D19 x D16	C5 (P + F_2)																		0.64	0.56			0.53	0.66		0.60	
NAC2-5A Root	C6 (M + F_2)																	0.56									
D16 x D19	C6 (P + F_2)																	0.62									
CKX activity Root	M + F2																										
	P + F2																										
CKX activity Spike	M + F2																										
	P + F2																										
Plant height	M + F2																						0.68	0.65			
D19 x D16	P + F2																						0.56	0.56			
Plant height	M + F2																							0.62		0.55	0.46
D16 x D19	P + F2																							0.69		0.76	0.66
Spike number	M + F2																						0.83	0.81			
D19 x D16	P + F2																						0.80	0.83			
Spike number	M + F2																					0.65	0.80	0.67	0.62		
D16 x D19	P + F2																					0.70	0.84	0.67	0.45		
Empty spike number	M + F2																										
D19 x D16	P + F2																										
Empty spike number	M + F2																										
D16 x D19	P + F2																										
Semi-empty spike numbe	M + F2																										
D19 x D16	P + F2																										
Semi-empty spike numbe	M + F2																										
D16 x D19	P + F2																										
Grain number	M + F2																							0.98	0.46	0.62	
D19 x D16	P + F2																							0.97	0.72	0.45	
Grain number	M + F2																							0.95	0.77		
D16 x D19	P + F2																							0.94	0.68		
Grain yield	M + F2																								0.38	0.75	
D19 x D16	P + F2																								0.61	0.63	
Grain yield	M + F2																								0.79		0.59
D16 x D19	P + F2																								0.72		0.42
Spike length	M + F2																										
D19 x D16	P + F2																										
TGW	M + F2																										
D19 x D16	P + F2																										
Root weight	M + F2																										
D19 x D16	P + F2																										

No data of CKX activity for C6

C7: D19 x KOH7 (M & F2; P & F2) C8: KOH7 x D19 (M & F2; P & F2)		*CKX1* Spike	*CKX2.1* Spike	*CKX2.2.2* Spike	*CKX5* Spike	*CKX9* (10) Spike	*CKX10* (9) Spike	*CKX11* (3) Spike	*NAC2-5A* Spike	*CKX1* Root	*CKX3* (6) Root	*CKX5* Root	*CKX8* (11) Root	*CKX10* (9) Root	*CKX11* (3) Root	*NAC2-5A* Root	CKX activity Root	CKX activity Spike	Plant height	Spike number	Empty spike number	Semi-empty spike number	Grain number	Grain yield	Spike length	TGW	Root wieght
CKX1 Spike	C7 (M + F_2)		0.69	0.62		0.61		0.33			0.50														0.64		
D19 x KOH7	C7 (P + F_2)		0.24	-0.08		-0.09		0.71			0.71														0.04		
CKX1 Spike	C8 (M + F_2)								0.81	0.84		0.82		0.51													
KOH7 x D19	C8 (P + F_2)								0.34	0.85		0.84		0.65													
CKX2.1 Spike	C7 (M + F_2)			0.85		0.76			-0.62																	-0.78	
D19 x KOH7	C7 (P + F_2)			0.53		-0.33			-0.18																	-0.75	
CKX2.1 Spike	C8 (M + F_2)			0.73								-0.60	-0.62										0.44		0.25	-0.50	
KOH7 x D19	C8 (P + F_2)			0.81								-0.42	0.15										0.72		0.69	-0.79	
CKX2.2.2 Spike	C7 (M + F_2)					0.68										-0.50										-0.64	
D19 x KOH7	C7 (P + F_2)					-0.22										-0.60										-0.46	
CKX2.2.2 Spike	C8 (M + F_2)						0.16	-0.70											-0.70							-0.71	
KOH7 x D19	C8 (P + F_2)						0.79	-0.51											-0.47							-0.85	
CKX5 Spike	C7 (M + F_2)					-0.09	0.53		0.67																	0.37	
D19 x KOH7	C7 (P + F_2)					-0.65	0.73		0.82																	0.64	
CKX5 Spike	C8 (M + F_2)								0.72		0.03				-0.73												
KOH7 x D19	C8 (P + F_2)								0.58		-0.67				-0.46												
CKX9 (10) Spike	C7 (M + F_2)						0.17								-0.07										0.60	-0.79	
D19 x KOH7	C7 (P + F_2)						-0.69								0.64										0.56	-0.31	
CKX9 (10) Spike	C8 (M + F_2)															0.79			-0.57								
KOH7 x D19	C8 (P + F_2)															0.77			-0.59								
CKX10 (9) Spike	C7 (M + F_2)																					0.70					
D19 x KOH7	C7 (P + F_2)																					0.53					
CKX10 (9) Spike	C8 (M + F_2)																				0.59	0.41				0.08	-0.69
KOH7 x D19	C8 (P + F_2)																				0.31	0.65				-0.72	-0.34
CKX11 (3) Spike	C7 (M + F_2)										0.68				0.76												
D19 x KOH7	C7 (P + F_2)										0.67				0.53												
CKX11 (3) Spike	C8 (M + F_2)																										
KOH7 x D19	C8 (P + F_2)																										
NAC2-5A Spike	C7 (M + F_2)												0.64												-0.16		-0.21
D19 x KOH7	C7 (P + F_2)												0.07												-0.79		-0.64
NAC2-5A Spike	C8 (M + F_2)									0.62		0.60	-0.01								0.59						
KOH7 x D19	C8 (P + F_2)									0.58		0.55	0.93								0.26						
CKX1 Root	C7 (M + F_2)																			-0.60			-0.61				-0.84
D19 x KOH7	C7 (P + F_2)																			-0.59			-0.59				-0.81
CKX1 Root	C8 (M + F_2)											0.86		0.60						-0.82			-0.75	-0.70			
KOH7 x D19	C8 (P + F_2)											0.86		0.49						-0.84			-0.67	-0.72			
CKX3 (6) Root	C7 (M + F_2)											0.75						0.94									
D19 x KOH7	C7 (P + F_2)											0.69						0.74									
CKX3 (6) Root	C8 (M + F_2)																										-0.76
KOH7 x D19	C8 (P + F_2)																										-0.72
CKX5 Root	C7 (M + F_2)															-0.63		0.74									
D19 x KOH7	C7 (P + F_2)															-0.55		0.81									
CKX5 Root	C8 (M + F_2)													0.60						-0.78		-0.66					
KOH7 x D19	C8 (P + F_2)													0.58						-0.78		-0.57					
CKX8 (11) Root	C7 (M + F_2)																			-0.39							
D19 x KOH7	C7 (P + F_2)																			-0.68							
CKX8 (11) Root	C8 (M + F_2)														0.60					-0.66		-0.72					
KOH7 x D19	C8 (P + F_2)														-0.18					-0.49		-0.45					
CKX10 (9) Root	C7 (M + F_2)																										
D19 x KOH7	C7 (P + F_2)																										
CKX10 (9) Root	C8 (M + F_2)																	0.70									
KOH7 x D19	C8 (P + F_2)																	0.44									
CKX11 (3) Root	C7 (M + F_2)																										
D19 x KOH7	C7 (P + F_2)																										
CKX11 (3) Root	C8 (M + F_2)																	0.40	0.63								
KOH7 x D19	C8 (P + F_2)																	0.65	-0.11								
NAC2-5A Root	C7 (M + F_2)																										
D19 x KOH7	C7 (P + F_2)																										
NAC2-5A Root	C8 (M + F_2)																					-0.52					
KOH7 x D19	C8 (P + F_2)																					-0.75					
CKX activity Root	M + F2																										
	P + F2																										
CKX activity Spike	M + F2																										
	P + F2																										
Plant height	M + F2																							0.71			
D19 x KOH7	P + F2																							0.63			
Plant height	M + F2																										
KOH7 x D19	P + F2																										
Spike number	M + F2																						0.86	0.85			
D19 x KOH7	P + F2																						0.90	0.86			
Spike number	M + F2																						0.79	0.77			
KOH7 x D19	P + F2																						0.68	0.76			
Empty spike number	M + F2																										
D19 x KOH7	P + F2																										
Empty spike number	M + F2																						-0.54				
KOH7 x D19	P + F2																						-0.64				
Semi-empty spike numbe	M + F2																										
D19 x KOH7	P + F2																										
Semi-empty spike numbe	M + F2																										
KOH7 x D19	P + F2																										
Grain number	M + F2																							0.92	0.75		
D19 x KOH7	P + F2																							0.94	0.45		
Grain number	M + F2																							0.98	0.29		
KOH7 x D19	P + F2																							0.84	0.71		
Grain yield	M + F2																										
D19 x KOH7	P + F2																										
Grain yield	M + F2																										0.45
KOH7 x D19	P + F2																										0.60
Spike length	M + F2																									-0.65	0.33
D19 x KOH7	P + F2																									-0.22	0.62
TGW	M + F2																										
D19 x KOH7	P + F2																										
Root wieght	M + F2																										
D19 x KOH7	P + F2																										

Table S2. Primer sequences designed for reference gene Ref 2, TaCKX GFMs and TaNAC2-5A

Ref 2	Ta2291R	NCBI	GCTTCTGCCTGTCACATACGC	165
	Ta2291F		GCTCTCCAACAACATTGCCAAC	
TaCKX1	TaCKX1_188R		CCCAGGTACTCCTTGTACCCTAT	188
	TaCKX1_188F		GTCTACCCGCTCAACAAATCC	
TaCKX2.1	TaCKX2_3_R_144		TCTCCTCGTTCTGCTCCTCC	144
	TaCKX2_3_F_144		TCTACCCCATGAACCGGGAC	
TaCKX2.2.1	TaCKX2_1_R_205		TATCACATACGCCATCCATGC	205
	TaCKX2_1_F_205		TTGATCGCGGAGCTAATCCA	
TaCKX2.2.2	TaCKX2_2_R_175		ATCGTATCCTGGCCTCCTCA	175
	TaCKX2_2_F_175		TACCCCATGAACCGGAACAG	
TaCKX3 (6)	TaCKX6_182R		CCGTGCTTGAATGTCTGC	182
	TaCKX6_182F		CACAAAGGAGGAGAAGGAGATG	
TaCKX4	TaCKX4_112_R		CTCCAAAGTCACACCCTCTACAC	112
	TaCKX4_112_F		AAGAACACGCAGCATAGCAAC	
TaCKX5	TaCKX5_3B_4R		CATACATGACACCAACGTACATCTT	150
	TaCKX5_3B_4F		GTCCGATTTTTGAGAAGACTGATT	
TaCKX8 (11)	TaCKX11_R_184		AGTCATGCACTGCAAACTCTATG	184
	TaCKX11_F_184		GACCAAGAGCTTCTGATCTCAAT	
TaCKX9 (10)	TaCKX10_R_167		ACATAAAGCAATTTACCTGGACTTG	167
	TaCKX10_F_167		GAGCTAAGGGCTTGTGGGA	
TaCKX10 (9)	TaCKX9_R_278		GTCCCTGTTCATGGGGTACA	278
	TaCKX9_F_278		CCACGGTGGATCAGAAGCTC	
TaCKX11 (3)	TaCKX3_150R		GAATTAGAGTTCACGGCTTGATG	150
	TaCKX3_150F		TTGTCAAGGGACTGTAGTAGGG	
TaNAC2-5A	TaNAC2_R		GATGATGGAGCCCAAGGCGGAG	100
	TaNAC2_F		CTGGGTGCTCTGCCGGCTCTAC	
